# Genomic Analysis of *Acinetobacter baumannii* Isolates Carrying OXA-23 and OXA-58 Genes from Animals Reveals ST1 and ST25 as Major Clonal Lineages

**DOI:** 10.3390/antibiotics11081045

**Published:** 2022-08-03

**Authors:** Lisa Jacobmeyer, Torsten Semmler, Ivonne Stamm, Christa Ewers

**Affiliations:** 1 Institute of Hygiene and Infectious Diseases of Animals, Department of Veterinary Medicine, Justus-Liebig University Giessen, 35392 Giessen, Germany; Lisa.Jacobmeyer@vetmed.uni-giessen.de; 2 NG1 Microbial Genomics, Robert Koch Institute, 13353 Berlin, Germany; SemmlerT@rki.de; 3 Vet Med Labor GmbH, 70806 Kornwestheim, Germany; Ivonne.Stamm@idexx.com

**Keywords:** companion animal, carbapanemase, OXA, international clone, resistance island, veterinary

## Abstract

*Acinetobacter baumannii* is increasingly being recognized as a relevant pathogen for animals with a putative zoonotic impact. This study aimed at identifying and characterizing carbapenemase-producing *A. baumannii* from animals. Among 503 *A. baumannii*, mainly isolated from dogs/cats (75.7%) between 2013 and 2018, 42 isolates from 22 veterinary clinics (VCs) harboured *bla*_OXA-58_ (*n* = 29) or *bla*_OXA-23_ (*n* = 13). The *bla*_OXA-58_ gene was located on plasmids (11.4–21.1 kb) within different genetic surroundings (patterns A–D). *Bla*_OXA-23_ was embedded in Tn*2006* on the chromosome (*n* = 4; pattern a) or Tn*2008* on plasmids (*n* = 9; 41.2–71.3 kb; patterns b–e). The predominant IC1-ST1^P^-OXA-58 (66.7%; 96.4% cgMLST complex type (CT)-1808) was disseminated among 11 VCs in Germany. Resistance islands *Aba*R3-like (*n* = 15) and *Aba*R10 (*n* = 1) have emerged among ST1-isolates since 2016. IC7-ST25^P^-OXA-23 isolates (21.4%) occurred in seven VCs in Germany, France and Italy and differed in their resistance gene patterns from those of OXA-58 isolates. They were separated into six CTs, basically according to their regional origin. Other STs observed were ST10, ST578 and ST602. In conclusion, OXA-23 and OXA-58 were linked with ST1 and ST25, two globally distributed lineages in humans. The suggested transmission of certain lineages within and among VCs together with the acquisition of *Aba*R islands hints at a successful dissemination of multidrug-resistant strains in the VC environment.

## 1. Introduction

*Acinetobacter baumannii* is an opportunistic Gram-negative bacterium that is responsible for a wide range of healthcare-associated infections such as ventilator-associated pneumonia, wound infections or catheter-associated bloodstream infections, especially in intensive care units (ICU) [1]. The majority of hospital-acquired infections are caused by *A. baumannii* strains belonging to international clones IC1, IC2 and, more recently, to IC7, corresponding to clonal complexes CC1, CC2, and CC25 and to sequence types ST1, ST2 and ST25 (Pasteur scheme) [1,2,3,4]. In the case of infections with multidrug-resistant *A. baumannii*, therapeutic options are generally limited. Of particular concern is the emergence of *A. baumannii* with resistance to carbapenems, as these are the cornerstone of treatment for *A. baumannii* infections [5]. Carbapenem-resistant (CR) *A. baumannii* are among the critical-priority pathogens on the WHO priority list of antibiotic-resistant bacteria for effective drug development [6]. In addition to the presence of chromosomally located *bla*_OXA-51_-like genes that confer carbapenem-resistance when overexpressed due to insertion sequences upstream of the genes, the acquisition of plasmid-located carbapenemases, predominantly oxacillinases OXA-23, OXA-58 and NDM-like β-lactamases, represent the most common mechanisms of CR in *A. baumannii* [1,7]. Carbapenemase (CP)-producing *A. baumannii* are on a global rise in humans, and in the last decade, a growing number of studies also confirmed their dissemination in companion [8,9,10,11,12,13] and livestock animals including horses [14,15,16,17,18]. Previous reports gave evidence that *A. baumannii* from companion animals revealed the same clonal lineages and resistance determinants as human strains, indicating a spill-over of such strains from humans to animals [8,11,19]. 

The aim of this study was to determine the presence of CP-producing *A. baumannii* isolates among clinical isolates from animals. CP producers were assessed for their antimicrobial susceptibility and their genomes were sequenced to identify resistance genes and their genetic surroundings and to determine the phylogenetic background of the isolates by multilocus sequence typing (MLST) and core genome MLST analysis. 

## 2. Results

### 2.1. Presence and Type of Carbapenemase Genes

We identified 42 isolates that carried a carbapenemase gene. Among the first collection of 473 randomly collected *A. baumannii* isolates, 15 (3.2%) isolates carried a CP gene: *bla*_OXA-58_ in all cases (Table 1). Nine of these isolates were from clinical specimens that had been taken from six dogs (one dog was sampled twice at an interval of five days) and two cats. Five isolates were cultivated from medical devices, i.e., from central venous catheters, that were used along with the treatment of another three dogs and two cats. Finally, one isolate was cultivated from an air conditioner in a university clinic for small animals by pure chance. Among the second collection (*n* = 29) of pre-selected carbapenem non-susceptible *A. baumannii* isolates from dogs and cats, 27 (93.1%) isolates carried acquired CP genes, namely *bla*_OXA-58_ (*n* = 14/48.37%) and *bla*_OXA-23_ (*n* = 13/44.8%). For reasons of clarity, we will refer to the CP-positive *A. baumannii* isolates from the first and second sample collection as one group of 42 isolates. The 42 CP positive strains of this study were obtained from 22 veterinary clinics and practices (referred to as clinic-1 to clinic-22) in Germany (31 isolates; 13 clinics), France (9 isolates; 7 clinics), and Italy (2 isolates; 2 clinics). with a maximum of nine CR *A. baumannii* isolates obtained from one institution, namely clinic 4 (JLU university clinic). The NDM-1-producing isolate has recently been published by our group and will not be further considered in the Results section [13].

### 2.2. MLST and Phylogenetic Analysis

The majority of CP-producing *A. baumannii* isolates belonged to the worldwide distributed sequence types ST1^P^/ST231^Ox^ (66.7%; isolated from dogs and cats in Germany) and ST25^P^/ST229^Ox^ (21.4%; isolated from dogs and cats in Germany, France, and Italy), representing international clones IC1 and IC7 (Table 2). Other STs determined were ST602^P^/ST732^Ox^ (7.1%; isolated from three cats in France), ST10^P^/ST447^Ox^ (2.4%; isolated from a dog in Italy), and ST578^P^/ST799^Ox^ (2.4%; isolated from a dog in France). While ST10^P^ belongs to IC8, the other two STs could not be grouped to IC1-IC9 based on a comparison of the genome of companion animal *A. baumannii* isolates with the genomes of representative members of international clones 1 to 9 (Supplementary Materials Figure S1).

**Figure 1:**
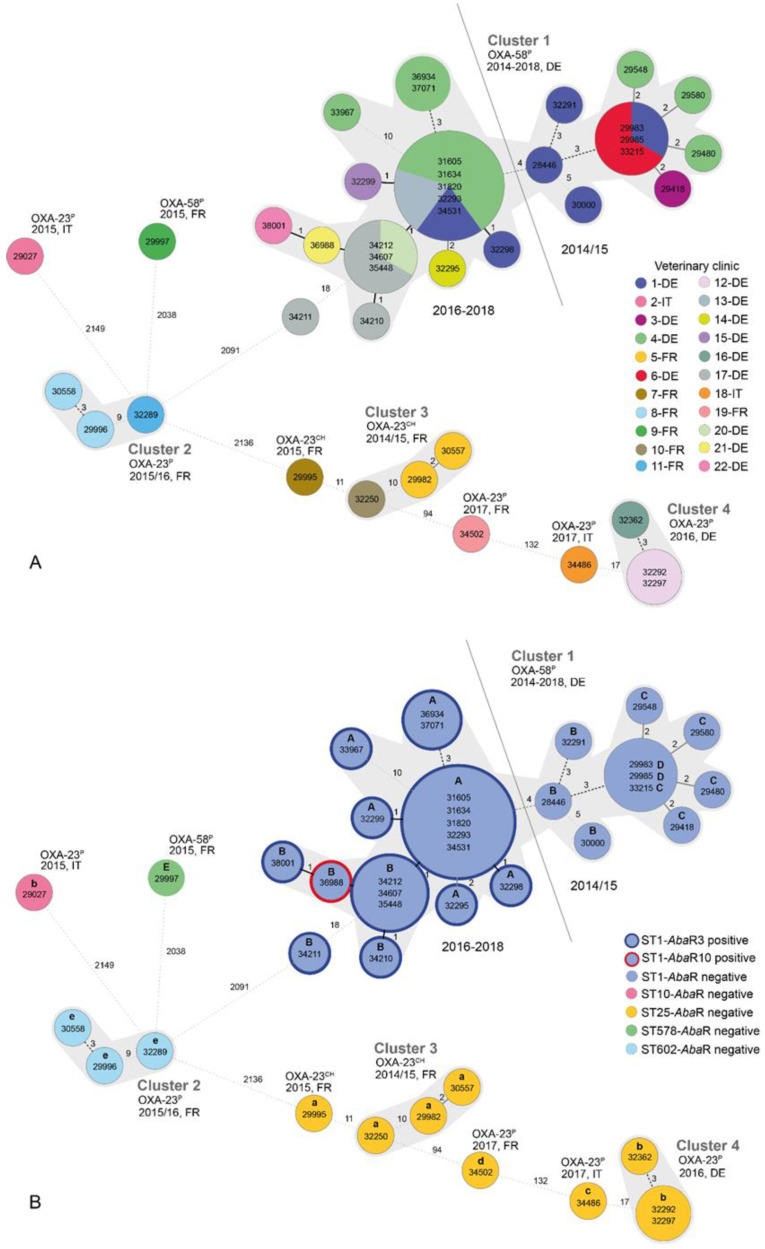
Minimum spanning tree based on cgMLST allelic profiles of 42 CP-producing *A. baumannii* isolates. (**A**) Distribution of cgMLST clusters with respect to the source of isolates regarding veterinary clinic and country (Germany = DE, France = F, and Italy = IT); (**B**) multilocus sequence type and presence of *A. baumannii* resistance islands. Each circle represents an allelic profile, i.e., complex type (CT), based on sequence analysis of 2390 target genes (pairwise ignoring missing values). The numbers inside the circles represent isolate designation. The numbers on the connecting lines illustrate the number of target genes with different alleles. Clonally related CTs (≤10 allele differences) are grey shaded and numbers of clusters are indicated. The presence of CPs OXA-23 and OXA-58 and their location (^P^ = plasmid; ^CH^ = chromosomal) as well as the year of strain isolation are indicated. Patterns of flanking regions of the *bla*_OXA-58_ (A–D) and *bla*_OXA-23_ regions (a–e) are given above the strain names in part B of the figure.

A minimum spanning tree based on cgMLST complex types (CT) of 42 CP-producing isolates was created (Figure 1). The cgMLST clustering was based on 2390 genes and broadly followed the grouping of the isolates into STs. As expected, cgMLST analysis revealed a higher resolution compared to the results from the seven-gene MLST. Twelve different cgMLST CTs were determined. Eight CTs were unique, the remaining four types were assigned to four clusters. With the exception of ST1 isolate IHIT34211 (CT-2175), that was cultivated from the nose of a dog from veterinary clinic-17 in Germany in 2017, the remaining 27 ST1 isolates belonged to the predominant complex-type CT-1808, given the species-specific threshold of ≤10 alleles differences. Accordingly, all these isolates belonged to a single cluster (cluster 1). They were restricted to Germany and were obtained from twelve veterinary clinics between December 2014 and August 2018 (Figure 1B, Table 2). Cluster 2 included three ST602 isolates (CT-1368 and CT-2191) from two veterinary clinics in France, and cluster 3 comprised three ST25 isolates (CT-2184 and CT-2186), also from two clinics in France. Finally, cluster 4 was made up of three ST-25 isolates (CT-2177) from two clinics in Germany. Overall, the ST25 isolates were assigned to six different CTs that differed by 2 to 132 alleles. 

**Figure 2:**
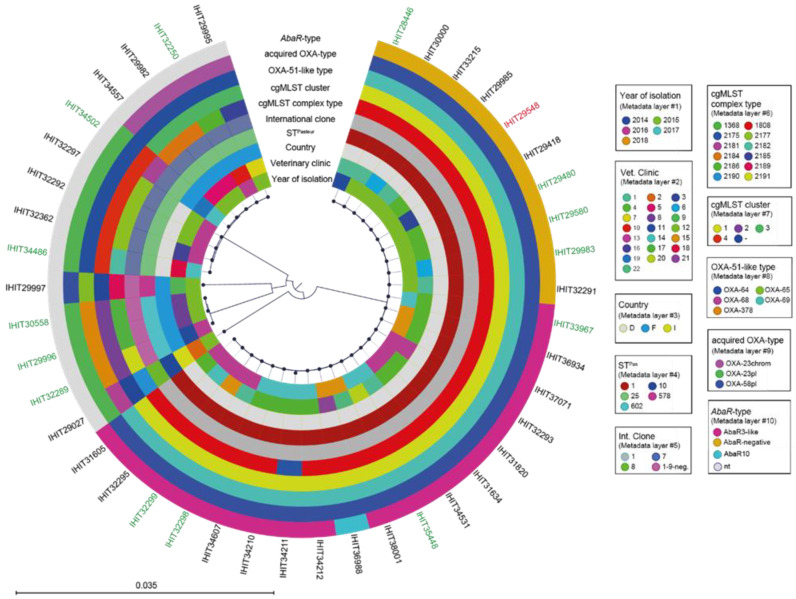
Maximum-likelihood tree of 42 genomes of carbapenemase-producing *A. baumannii* isolates. The phylogeny is based on 2,646 orthologous genes and was calculated by RAxML v8. Graphical visualization was achieved by iTOL v5. Colored rings represent (from inner to outer ring the (**i**) year of isolation, (**ii**) veterinary clinic, (**iii**) country of isolation, (**iv**) multilocus sequence type (Pasteur scheme), (**v**) international clone, (**vi**) core genome MLST complex type, (**vii**) core genome MLST cluster, (**viii**) OXA-51-like type, (**ix**) acquired oxacillinase type, and (**x**) *A. baumannni* resistance island type. Color of strain numbers indicates the origin of the isolates (green = cats; black = dogs; red = environment).

A phylogenomic tree of all strains sequenced in the present study including relevant metadata is provided in the following figure (Figure 2). 

### 2.3. Genomic Location of Carbapenemase Genes and Ttransformation Assays

**Figure 3:**
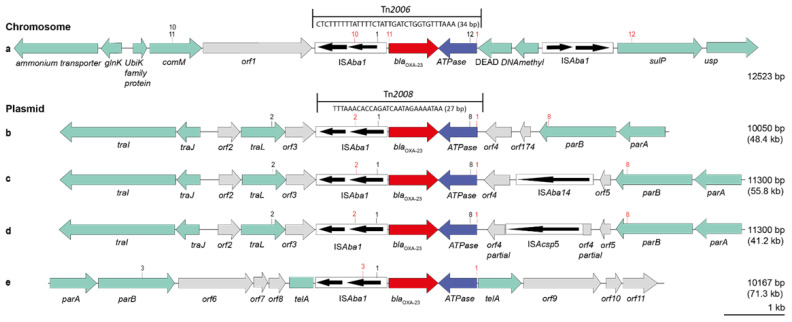
Genetic environment of *bla*_OXA-23_ genes in 13 *A. baumannii* from dogs and cats. (**a**): Chromosomally located *bla*_OXA-23_; (**b**–**e**): *bla*_OXA-23_ located on plasmids (partially shown).

**Figure 4:**
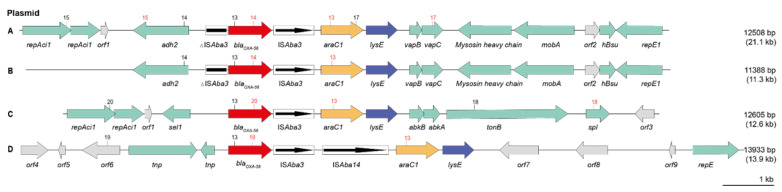
Genetic environment of *bla*_OXA-58_ genes in 29 *A. baumannii* isolates from dogs and cats. (**A**–**D**) *bla*_OXA-58_ located on different plasmids. Extent and orientation of genes and open reading frames (*orf*) are shown by a horizontal arrow with the gene names below. The *bla*_OXA-58_ gene is shown in red, *araC1* in yellow, *lysE* in blue, *orfs* in grey and other genes in light green. Insertion sequence (IS) elements are represented by a black framed box with the orientation of the transposase genes shown within. Primer pairs used for bridging PCRs are shown as numbers (forward primer in black, reverse primer in red) above the respective genes (Supplementary Materials Table S3). Numbers to the right indicate the size of the genetic regions displayed in the figure and the estimated total size of the plasmids (shown in brackets).

Southern blot hybridization of S1-Nuclease digested whole-cell DNA together with whole-genome sequence data analysis revealed the location of *bla*_OXA-23_ and *bla*_OXA-58_ on plasmids for 38 *A. baumannii* isolates. The remaining four isolates, all ST25 (CT-2184, CT-2185, and CT-2186) and all from one veterinary clinic in France, carried their *bla*_OXA-23_ gene on the chromosome (Figure 3, pattern a). Data from bridging PCRs and amplicon sequencing revealed the presence of four different OXA-23 plasmids, varying from 41.2 kb (plasmid type d, 1 isolate) to 48.4 kb (b, 4 isolates), 55.8 kb (c, 1 isolate) and 71.3 kb (e, 3 isolates) in size. OXA-58 plasmids appeared in four different types with 21.1 kb (plasmid type A, 11 isolates), 11.3 kb (B, 10 isolates), 12.6 kb (C, 7 isolates), and 13.9 kb (D, 1 isolate) in size. None of the plasmids carried other antimicrobial resistance genes. The direct genetic environment of *bla*_OXA-23_ and *bla*_OXA-58_ genes in the different plasmid types is illustrated in Figure 3 and Figure 4, and the assignment of isolates to plasmid types is provided in Table 2. 

Distinct plasmid types were basically linked with a certain ST and CT, but distinct STs and CTs could also reveal the presence of different plasmid types. For example, OXA-58 plasmid type A appeared solely in ST1/CT-1808 isolates. However, ST1/CT-1808 isolates carried not only OXA-58 plasmids of type A, but also of types B, C, and D. In contrast, OXA-23 plasmids were not detected among ST1/CT-1808 isolates but were distributed among ST10, ST25 and ST602 isolates in Germany, Italy and France. Notably, OXA-23 plasmid type b was detected in three ST25 isolates from Germany and in one ST10 isolate from Italy. Regarding this exception, OXA-23 and OXA-58 plasmid types differed according to STs and country of origin. Veterinary clinics where at least two CP-producing isolates were obtained mostly revealed more than one plasmid type, i.e., clinic 1 (OXA-58 plasmid type A, B and D), clinic 4 (type A and C) and clinic 6 (group C and D) (Table 2). 

A total of 24 of the 38 CR-*A. baumannii* with plasmid-located OXA-23 (*n* = 8) and OXA-58 (*n* = 16) CP genes, representing different MLST types, animal hosts and isolation dates, were included in transformation assays. Except for three OXA-23 plasmids, the OXA-23/OXA-58 plasmids of the remaining 21 isolates were transformable. Compared with the *A. baumannii* recipient strain ATCC 17978, the transformants showed increased MICs for piperacillin (≥128 mg/L; 100% resistant) and imipenem (2–≥16 mg/L; 63% resistant) (Supplementary Materials Table S1).

### 2.4. Resistance Phenotype and Genotype

All 42 CP-producing isolates were resistant to imipenem (MIC ≥ 8 mg/L) (Supplementary Materials Table S2). According to the results from E-Test, 27 isolates were resistant to meropenem (MIC from 8 to ≥32 mg/L) and 21 isolates were resistant to doripenem (MIC from 6 to ≥32 mg/L). All isolates showed resistance (either acquired or intrinsic) to ampicillin, piperacillin, piperacillin/tazobactam, amoxicillin/clavulanate, cephalotin, cephalexin, chloramphenicol, tetracycline, and nitrofurantoin. In addition, a different number of strains was resistant to cefpirom (23.8%), gentamicin (54.8%), ciprofloxacin (90.5%), moxifloxacin (88.1%), enrofloxacin (90.5%), marbofloxacin (90.5%), and to the sulfamethoxazole–trimethoprim combination (59.5%). Colistin MICs ranged from 0.5 to 2.0 mg/L (all susceptible), while low MICs were observed for tigecycline (S MIC ≤ 0.5–1 mg/L, 100%), amikacin (S ≤ 2 mg/L, *n* = 40, 95.2%; *I* = 32mg/L, *n* = 2, 4.8%) and tobramycin (S ≤ 1 mg/L, *n* = 41; 97.6%; *I* = 8 mg/L, *n* = 1, 2.4%). 

Multiple antimicrobial-resistance (AMR) genes and profiles were detected in the CP-producing *A. baumannii* isolates. We identified β-lactamase genes *bla*_ADC-2_-like (2.4%), *bla*_ADC-11_ (66.7%), *bla*_ADC-26_ (21.4%), *bla*_ADC-32_ (7.1%), *bla*_ADC-76_ (2.4%), and *bla*_TEM-1_ (42.9%), aminoglycoside modifying enzymes *aac(3)-Ia* (40.5%), *aac(3)-IIa* (11.9%), *aad(3`)-IVa* (2.4%), *aac(6`)-Ian* (11.9%), *aadA1* (40.5%), *aph(3`)-Ia* (2.4%), *aph(3`)-VIa* (4.8%), *aph(3``)-Ib* (21.4%), and *aph(6)-Id* (21.4%), tetracycline genes *tet*(A) (40.5%), *tet*(B) (14.3%) and *tet*(R) (54.8%), sulphonamide-resistance genes *sul1* (42.9%) and *sul2* (14.3%), chloramphenicol acetyltransferase gene *catA1* (40.5%), and trimethoprim-resistance gene *dfrA1* (2.4%). In addition, the distribution of antibiotic efflux pump genes was as follows: *abeS*, *abaF*, *abaQ*, *amvA*, *adeF*, *adeJ*, *adeK*, and *adeL* (100% each), *adeC* (69.0%), *adeG* (73.8%), *adeH* (69.0%), *adeI* (78.6%), *adeN* (88.1%), and *adeR* (97.6%). The disinfectant efflux pump gene *qacE∆1* was present in 40.5% of the isolates. *Acinetobacter baumannii* antibiotic resistance islands (*Aba*R) were detected in 64.3% of the 28 ST1 isolates, whereas isolates belonging to ST10, ST25, ST578 and ST602 were *Aba*R-negative. Notably, ST1 isolates from earlier than 2016 were also *Aba*R-negative, while all isolates obtained since 2016 carried either an *Aba*R3-like structure (*n* = 17) or *Aba*R10 (*n* = 1; isolate from 2018) (Supplementary Table S2; Figure 1B). As originally described, the *Aba*R3-like island consisted of a Tn*6019* backbone, was embedded in the chromosomal ATPase gene *comM* [20,21] and carried a multiple antibiotic resistance region (MARR) in the center in transposon *Tn*6018 containing *bla*_TEM-1_, *aacC1*, *aadA1*, *tet*(A), *tet*(R), *sul1* and *catA1*. In contrast to the original *Aba*R3 island, which is 63 kb in size in the human IC1-*A baumannii* strain A85 [22], the *Aba*R3-like island lacked the *Tn*6020-related gene *aphA1* and revealed a disruption in the IS*Aba1* sequences that are usually flanking both sides of the kanamycin gene. As previously described for strainAB058, the *Aba*R10 island was 31 kb in size and carried only *sul1* in the MARR of Tn*6018* [23]. OXA-58 *A. baumannii* strains assigned to cgMLST CT-1808 showed a very similar profile of efflux systems. All CT-1808 isolates that carried the *Aba*R3-like island also carried efflux genes of the major facilitator superfamily MFS (*abaF*, *abaQ*, *amvA* and *qacE∆1*), the resistance-nodulation-cell-division (RND) family (*adeC*, *adeF*, *adeG*, *adeH*, *adeI*, *adeJ*, *adeK*, *adeL*, *adeN* and *adeR*) and the small multidrug resistance (SMR) family (*abeS*). The *Aba*R10 positive strain and *Aba*R-negative strains of CT-1808 revealed the same efflux gene profile but lacked *qacE∆1*. Only one *bla*_OXA-58_ strain assigned to ST578/CT-2189 showed a unique efflux gene profile (*abeS*, *abaF*, *abaQ*, *amvA*, *adeF*, *adeH*, *adeI*, *adeJ*, *adeK*, *adeL*, *adeN* and *adeR*). None of the *bla*_OXA-23_ isolates carried either *qacE*∆1 or *adeH*. Strains of the same ST also showed a similar efflux gene profile, except for one ST25 strain, that did not possess *adeN* (Supplementary Materials Table S2).

All 42 isolates carried intrinsic oxacillinases that correlated well with the sequence types of the isolates: ST1^P^ - OXA-69, ST25^P^ - OXA-64, ST10^P^ - OXA-68, ST578^P^ - OXA-65, and ST602^P^ - OXA-378. Only one ST25 strain, which was isolated from the urine of a cat from France (IHIT34502), revealed the presence of insertion sequence IS*Aba1* upstream of the oxacillinase gene *bla*_OXA-64_ and also upstream of its *bla*_ADC-26_ gene.

### 2.5. Flanking Region of Carbapenemase Genes

To characterize the *bla*_OXA-23_ genomic region in the different strains, WGS contigs of 175 bp to 57,862 bp in size were used to create primer pairs for PCR mapping. In the case of *bla*_OXA-58_ plasmids, one to ten contigs between 74 bp and 21,069 bp per isolate were concatenated in silico based on PCR mapping results.

The transposons Tn*2008* and Tn*2006* were identified as genetic structures harbouring the *bla*_OXA-23_ gene (Figure 3). In Tn*2008*, the *bla*_OXA-23_ gene was flanked by the insertion sequence IS*Aba1* 27 bp upstream, and by a putative *ATPase* gene downstream. *Tn*2006 is similar to *Tn*2008, but here, the *bla*_OXA-23_ gene is flanked by two copies of IS*Aba1*, which are located in opposite orientations. In our isolates, the transposon structures Tn*2008* and Tn*2006* were embedded in different genomic regions (a–e), as shown in Figure 3 and Figure 1B. The *bla*_OXA-58_ genes were located on four different plasmid structures (A–D), as shown in Figure 4 and indicated in Figure 1B. 

The extent and orientation of genes and open reading frames (*orf*) are shown by a horizontal arrow with the gene names below. The *bla*_OXA-23_ gene is shown in red, the ATPase gene in blue, *orfs* in grey, and other genes in light green. Insertion sequence (IS) elements are represented by a black framed box with the orientation of the transposase genes shown within. Transposons Tn*2006* and Tn*2008* are shown above with an arrow indicating their location. Sequences below the transposon labels indicate the distance between IS*Aba1* and the *bla*_OXA-23_ start codon. Primer pairs used for bridging PCRs are shown as numbers (forward primer in black, reverse primer in red) above the respective genes (Supplementary Materials Table S3). Numbers to the right indicate the size of the genetic regions displayed in the figure and the estimated total size of the plasmids (shown in brackets). 

## 3. Discussion

*A. baumannii* is a globally distributed pathogen associated with clinical infections in both humans and animals. The emergence of multidrug-resistant isolates drastically limits antibiotic treatment options, particularly in intensive-care medicine [24]. Data on the antimicrobial resistance and phylogenetic background of *A. baumannii* isolates from diseased animals are quite limited. Only a few studies systematically screened a representative number of *A. baumannii* isolates for carbapenemases that are most common among humans, and even less made use of whole genome sequence analysis to provide a detailed characterization of the isolates [8,9,13,16,19,25]. 

In this study, we provide a comprehensive analysis of CP-producing *A. baumannii* isolates from animals that were either hospitalized or treated in veterinary clinics in Germany (*n* = 13 clinics), France (*n* = 7) and Italy (*n* = 2) during the years 2014 and 2018. We could show that OXA-23 and OXA-58, which are globally distributed CPs in humans, are frequently present in *A. baumannii* isolates obtained from clinical samples of cats and dogs and from medical devices used for the treatment of these animals. In contrast, none of the *A. baumannii* isolates from livestock animals (*n* = 50 isolates), horses (*n* = 46), small animals (*n* = 15), and other animals (*n* = 10) harboured a carbapenemase. To date, to the best of our knowledge, merely 12 studies described the occurrence of CPs, predominantly OXA-23, but also OXA-72 and NDM-1, in *Acinetobacter baumannii* from cats and dogs in Germany, France, Portugal, Italy, Serbia, Pakistan and Thailand [8,9,10,11,12,13,19,25,26,27,28] (Table 3). 

Infrequent findings of CP-producing isolates that were clearly assigned to the species *A. baumannii* have been reported from livestock animals, i.e., from cattle [15,31], sheep [18] swine [14,15,32], and poultry [15] from France, Croatia, Lebanon, Pakistan, and Canada. OXA-23 was the most frequently found CP in these studies while OXA-40, OXA-58, NDM-1 and PER-1 were single findings. 

We determined OXA-58 as the predominant CP type (69.1%), while about one third of the CP-producing isolates in our study carried the *bla*_OXA-23_ gene. Of note, nearly all OXA-58 isolates (96.6%) were from companion animals in Germany, while the majority of OXA-23 isolates (76.9%) were from companion animals in France and Italy, indicating a country-specific distribution. On the other hand, more than two thirds (69.2%) of the OXA-23 isolates were assigned to ST25, whereas OXA-58 was almost (96.6%) restricted to ST1 isolates. 

In Europe and also worldwide, OXA-23 is by far the most common β-lactamase among *A. baumannii* strains from human patients, including those involved in hospital outbreaks [7,33,34,35,36,37]. In Germany, the National Reference Laboratory for multidrug-resistant Gram-negative bacteria rated OXA-23 as predominant and OXA-72 as the second most common CP among *A. baumannii* isolates that were sent by nationwide microbiology laboratories for clarification of phenotypic carbapenem-resistance over the last years [38]. In contrast, OXA-58 was found in significantly lower numbers ranking between the third and tenth position between 2015 and 2020 but was not detected in 2021. The fact that 11 of 13 German veterinary clinics revealed the presence of OXA-58-producing isolates suggests a different distribution of this CP type in companion animals compared to what is going on in the medical field in our country. While this could indicate different sources of infections in humans and animals, a spillover of OXA-58 plasmids from humans to animals can still not entirely be ruled out. 

As the predominant ST among CP-producing *A. baumannii* in humans is ST2^Pa^ and our OXA-58 isolates all belonged to ST1 and almost always to cgMLST cluster type CT-1808, a transmission of isolates from humans to animals is less likely than an initial transfer of an OXA-58 plasmid by horizontal gene transfer and a subsequent clonal dissemination of CT-1808 among companion animals in Germany. 

The random finding of an OXA-58-producing *A. baumannii* isolate from an air conditioner in one of the veterinary clinics underlines the relevance of the clinical environment as a putative source of MDR bacteria. This has recently been shown for high-risk clone ST11 of CP-producing *Klebsiella pneumoniae* in a veterinary referral hospital in Switzerland. Brilhante et al. (2021) showed that clinical CP *K. pneumoniae* strains were highly related to those contaminating the clinical environment [39]. The strain from an air conditioner in the present study belonged to the most common cgMLST type 1808 and carried the same *bla*_OXA-58_ plasmid group C as the clinical strains detected in the same veterinary clinic. These results highlight the probability that MDR *A. baumannii* survive in the clinical environment and may be further dispersed and transferred to animals and humans.

Regarding France and Italy, where isolates from dogs and cats nearly always carried OXA-23, the situation appeared much more similar to that in humans in those countries. In a cross-sectional countrywide survey on the distribution of carbapenem-resistant *A. baumannii* in Italy, Principe et al. (2014) reported that among 25 centers, OXA-23 was the predominant CP (81.6%) followed by OXA-58 (4.5%), indicating an epidemic diffusion of OXA-23 [34]. Jeannot et al. (2014) determined *bla*_OXA-23_ in 82% of the samples that were collected along with 37 outbreaks in France, while *bla*_OXA-58_ was only present in 7% of the isolates [40]. Our group previously detected OXA-23 from an ST1^P^ isolate originating from a cat with urinary tract infection treated in clinic 4 of the present study in the year 2000 [8,9]. This isolate belonged to cgMLST type CT-720 and revealed up to 84 different alleles to CT-1808, suggesting that it is not a direct ancestor of this epidemic lineage. In addition, we recently described OXA-23 strains isolated from two dogs treated in veterinary clinics that are not included in the current study [8]. The two isolates belonged to ST10^P^/585^Ox^ and thus to the same ST that has been observed for one OXA-23 isolate of the present study, which originated from a dog in Italy (IHIT29027; ST10^P^/447^Ox^). However, the strain from Italy (CT-2190) and previous isolates from Germany (CT-717) differed in their cgMLST types by 425 alleles, indicating only moderate relatedness from a whole genome sequence perspective. As the fraction of animal isolates from France and Italy was quite low in the present study, conclusions should be considered with caution. 

The majority of our OXA-23 isolates (69.2%) belonged to ST25, which is a globally distributed clone that has been associated with infections and outbreaks in humans, particularly in the ICUs, in Europe, South America, and Asia [3,41,42]. Nine ST25 isolates were separated into six CTs and revealed three antimicrobial resistance patterns, underlining the phylogenetic and genomic diversity of the ST25 lineage as previously shown for human clinical isolates [3]. In Germany, CT-2177 seems to be established in the veterinary environment, while the strains from France showed from 2 to 94 allele differences, indicating an independent spread of different CTs. In 2017, Lupo and co-workers reported that OXA-23-ST25 *A. baumannii* might be endemically distributed in companion animals in France [19]. They investigated 41 *A. baumannii* isolates from diseased pets from 2011 to 2015 and determined seven OXA-23-producing *A. baumannii* that belonged to ST25. As the seven isolates originated in five departments in two regions, the authors suggested a clonal dissemination among companion animals in France. In support of this, Herivaux and co-workers identified a high rate of asymptomatic dog carriers of ST25-OXA-23-*A. baumannii* isolates (2.7%) in France in 2015 and suggested that pets could serve as a possible reservoir of community acquired infections [12]. Notably, in Switzerland, ST25 and ST1 are also the predominant *A. baumannii* lineages in hospitalized animals, although OXA-23 and OXA-58-producing isolates have not been described [43]. 

In a previous survey on *A. baumannii* isolates obtained from diseased companion animals in Germany between 2000 and 2013, we could show that ST2 was the major lineage among carbapenem-susceptible isolates. A very recent report from the Netherlands described two unrelated ST2 lineages of carbapenem non-resistant *A. baumannii* as a cause of two outbreaks in a companion animal ICU that occurred in 2012 and 2014. This, together with sporadic findings of carbapenemase-producing ST2-*A. baumannii* isolates in companion animals in Portugal [11], Italy [25], Thailand [26] and Pakistan [28], underlines that, with ST2, ST1 and ST25, three of the most relevant sequence types globally distributed in the human domain are also disseminated among companion animals. In a recent study from Germany, Schleicher et al. demonstrated that the population structure of carbapenem-susceptible *A. baumannii* from humans is highly diverse, whereas imipenem non-susceptibility was strongly linked with the clonal lineages IC2 and IC1, suggesting a high clonality of carbapenem-resistant isolates [44].

According to cgMLST analysis, the 42 carbapenem-resistant *A. baumannii* isolates were assigned to 12 cluster types. Five of these CTs were assigned to four clusters (cluster 1–4), while the remaining seven CTs were unique. As already mentioned, CT-1808 was predominant in Germany, where it occurred in 11 veterinary clinics. The repeated finding of this type over longer periods in VCs 1 (*n* = 6; 2014–2017) and 6 (*n* = 9; 2015–2018) underlines a putative nosocomial spread within these clinics. Most notably, earlier ST1/CT-1808 isolates from 2014 and 2015 (*n* = 10) only possessed cephalosporinase gene *bla*_ADC-11_ in addition to their *bla*_OXA-58_ gene. Later isolates collected between 2016 and 2018 have acquired either an *Aba*R3-like island (*n* = 16) or *Aba*R10 (*n* = 1) (Figure 1B). At least for clinic 1 and clinic 4, which yielded carbapenem-resistant ST1 isolates in both time periods, the emergence of *Aba*R3-like positive isolates could have occurred within the clinics, while the original entry of this resistance island into the population remains unsolved. Along with the uptake of the *Aba*R3-like island, the isolates gained resistance genes *tet*(A), *tet*(R), *sul1* and *catA1*, and the cassette-associated *aacC1* and *aadA1* genes (Supplementary Materials Table S2). Accordingly, they showed phenotypic resistance to gentamicin and to the trimethoprim–sulfamethoxazole combination and higher MICs (≥16 mg/L) to tetracycline as compared to the earlier ST1 isolates. Additionally, *Aba*R10, which was first described by Adams et al. (2010), rendered isolate IHIT36988 additional resistance to the trimethoprim-sulfamethoxazole combination as it encodes the *sul1* gene [23]. 

To date, the majority of human clinical IC1 isolates carry either *Aba*R0 or its derivative *Aba*R3, which arose around 1990, or a variant from one of them, that has evolved since then [22,45]. *Aba*R3 is a large genomic-resistance island within a complex transposon that is located at a specific position in the ATPase-encoding *comM* gene, which is involved in natural transformation of *A. baumannii* [21,46,47]. Previous variants arose by different mechanisms, including recombination events, gene cassette addition or replacement, and what also happened in our isolates: deletions caused by one of three internal IS*26* sequences [22,45]. We could show that complex *A. baumannii* resistance islands are not only globally distributed in IC1-*A. baumannii* isolates from humans but that they occur frequently among veterinary isolates and may undergo evolutionary changes. By that, the isolates might gain a selective advantage towards a certain antimicrobial selective environment in a VC, similar to what has happened in the medical field. Here, the selective advantage provided by *Aba*R3 or its variants could have played a role in the spread of IC1 in European hospitals in the 1980s, as the resistance mechanisms encoded by *Aba*R3 were effective against many antibiotics used at that time [46]. Most notably, a number of other variants of the *A. baumannii* resistance island arisen since then confer resistance to critical important antibiotics including fluoroquinolones, cephalosporins, and aminoglycosides [22,45] and thus contribute to the emergence of multi-drug-resistant strains [21,22,45].

All 29 OXA-58 positive isolates carried *bla*OXA-58 on a plasmid, almost always embedded in a previously described genetic context in human isolates with IS*Aba3*, *araC* and *lysE* [48,49]. However, when determining the wider environment, we identified four different patterns (A–D) around the *bla*_OXA-58_ gene, indicating different origins and evolutions of plasmids or genetic fragments. The patterns were always identical among isolates of the same complex-type but were not restricted to one CT. This could suggest both a clonal distribution of plasmids but also a spread by horizontal gene transfer. The genetic contexts of *bla*_OXA-58_ genes in our isolates shared high similarity with plasmids from human patients, such as pAba3207a of *A. baumannii* 3207 that was detected in Mexico [50], pWA3 of *A. baumannii* WA3 isolated in China [51], and pABIR of *A. baumannii* ABIR found in a patient in the Lebanon [52]. Tn*2008* of the OXA-23-positive *A. baumannii* isolates from our study shared high similarity (99.96%) with the same genetic region that was determined in *A. baumannii* isolate IHIT7853 (ST1, CT-720), which was isolated from a cat treated in VC4 in 2010 [8]. Furthermore, *A. baumannii* DU202, detected in a patient in Korea [52] and *A. baumannii* K50 that was isolated from a wound in a human patient in Kuwait, showed 99.9% identity with the transposon structure of our *bla*_OXA-23_-positive *A. baumannii* isolates [53]. This, together with the sharing of clonal lineages of *A. baumannii* isolates maintaining the OXA-23 and OXA-58 plasmids, suggests at least partial overlaps between certain groups of carbapenemase-producing *A. baumannii* isolates from humans and animals. High-resolution genomic and plasmidomic analysis including combined short- and long-read sequencing techniques will help to further elucidate the similarities and differences between these bacteria in the different populations. It may also help to infer whether transmission events could have occurred between humans and animals and in which direction they have taken place. 

## 4. Materials and Methods

### 4.1. Bacterial Strains and DNA Isolation

*Acinetobacter baumannii* strains were collected during routine microbiology investigations in two veterinary diagnostic laboratories in Germany. The first collection of 473 strains was isolated from clinical specimens (*n* = 398) or from different organs during necropsy (*n* = 40) obtained from dogs/cats (*n* = 351), livestock animals (*n* = 50), horses (*n* = 46), pets (*n* = 15), zoo animals (*n* = 7), and birds (*n* = 3) (Table 4). The samples were provided by 85 veterinary clinics and practices and by a university Institute of Veterinary Pathology in Germany between January 2013 and August 2018. Another 34 isolates of this first collection were obtained from clinical devices, such as catheter, tubes, and implants, used during therapeutic or surgical treatment of dogs and cats in three veterinary clinics; one strain was isolated from an air conditioner in a veterinary hospital. The 398 *A. baumannii* strains (all from Germany) from clinical specimens were isolated from wounds and abscesses (31.4%), respiratory tract and nasopharynx (21.1%), skin and hair (13.1%), the urinary tract (10.6%), and from various other body sites, as detailed in Supplementary Table S1. 

The second collection included carbapenem non-susceptible (imipenem MIC ≥ 2 mg/L) *A. baumannii* (*n* = 29) strains provided by an external veterinary diagnostic laboratory in Germany (Table 4). These isolates were collected from the urinary tract (*n* = 14), wounds (*n* = 7), and from other clinical sites from dogs (*n* = 19) and cats (*n* = 10) in veterinary clinics in Germany (*n* = 18), France (*n* = 9), and Italy (*n* = 2). 

All samples were initially cultivated on blood agar plates without antibiotics. Species identification was performed with MALDI-TOF MS using a Microflex LT/SH mass spectrometer and the biotyper database (Maldi Biotyper 3.0, V. 7.0) (both from Bruker Daltonics, Bremen, Germany) and by performing *gyrB* multiplex PCR [53]. 

To extract genomic DNA, late log-phase cells were harvested and lysed with EDTA, lysozyme, and detergent treatment, followed by proteinase K and RNase digestion using the DNeasy Blood & Tissue Kit (Qiagen, Hilden, Germany) according to the manufacturer’s recommendation. The DNA was stored at −20 °C until further use.

### 4.2. Detection of Carbapenemase Genes

*Acinetobacter baumannii* strains were screened for β-lactamase genes coding for CPs of the families OXA-23/-40/-58/-143/-235, VIM, NDM, KPC, OXA-48, GES, GIM, and IMP by PCR [54,55,56,57,58,59] using previously published primers and protocols (Supplementary Materials Table S4). The genomes of strains that possessed an acquired CP gene were further sequenced.

### 4.3. Antimicrobial Susceptibility Testing

*A. baumannii* strains that carried an acquired CP gene were tested for their antimicrobial susceptibility to 27 antimicrobial agents by using the VITEK 2 system (bioMérieux→, Nuertingen, Germany; AST cards GN38 and GN238). Antibiotic gradient strips (Liofilchem^®^, Roseto degli Abruzzi, Italy) were additionally used to test the susceptibility of the strains to imipenem, meropenem and doripenem. Susceptibility to colistin was determined by using the E1-102-040 plate of the MICRONAUT system (Merlin Diagnostics, Bornheim-Hersel, Germany). MICs were interpreted according to breakpoints defined for human *Acinetobacter* spp. by CLSI (Clinical and Laboratory Standards Institute. Performance standards for antimicrobial susceptibility testing. 29th ed. Document M100S.Wayne, PA: CLSI; 2019). For antibiotics without a defined breakpoint for *Acinetobacter* spp., breakpoints of agents belonging to the same antibiotic class or breakpoints for Enterobacterales were used for interpretation.

### 4.4. Genome Sequencing and Annotation

Sequencing of *A. baumannii* genomes was performed using Illumina MiSeq 300 bp paired-end sequencing with an obtained coverage > 90×. After quality control using the NGS tool kit13 (70% of bases with a phred score > 20), high-quality filtered reads were used for de novo assembly into contiguous sequences (contigs) and subsequently into scaffolds using SPAdes v3.9 (http://bioinf.spbau.ru/spades, accessed on 8 February 2022) [26]. Assembled draft genomes were annotated using Prodigal [27]. 

### 4.5. MLST, cgMLST and Phylogenetic Comparison

Multilocus sequence types were deduced from whole genome sequence data using MLST 2.0 (https://cge.cbs.dtu.dk/services/MLST/, accessed on 10 February 2022) and the MLST scheme developed by the Pasteur Institute [60]. To determine the clonal relationship of CR-*A. baumannii* strains, core genome MLST (cgMLST) implemented in Ridom SeqSphere + version 7.7.0 (Ridom GmbH, Germany) was performed [61]. Allelic profiles were compared by using the “pairwise ignoring missing values” parameter during distance calculation. The cluster definition threshold was set at a maximum difference of 10 alleles in a pairwise comparison. 

Phylogenetic relationships were determined by applying a gene-by-gene approach on the dataset to generate a core genome alignment and subsequently a phylogenetic tree. The core genome alignment was assembled by a gene-wise alignment with Mafft v7.407 (http://mafft.cbrc.jp/alignment/server/, accessed on 2 June 2022) of 2646 core genes that were present in at least 99% of the strains (sequence similarity min. 70%, sequence coverage min. 90%) and were concatenated afterwards. The resulting alignment was used to infer a phylogeny with 100 bootstrap replicates using RAxML v.8.2.10 (https://github.com/stamatak/standard-RAxML, accessed on 2 June 2022) with a general time-reversible model and gamma correction for site rate variation. iTOL v5 (https://itol.embl.de/, accessed on 2 June 2022) was used to visualize the population structure in the context of available metadata.

### 4.6. Identification of Antimicrobial Resistance Genes and Islands and of Genetic Regions Flanking Carbapenemase Genes

Services provided by the Center of Genomic Epidemiology (https://cge.cbs.dtu.dk/services/, accessed on 10 February 2022) were used to identify resistance genes (ResFinder v4.1). To investigate the genetic region surrounding CP genes, contigs that contained such genes were first compared with public sequences by BLASTn analysis, using Geneious v. R8.1 (Biomatters Ldt, Auckland, New Zealand). The same contigs and all remaining contigs of an isolate were then mapped to the identified reference sequences using the “Map to reference tool” of Geneious v R8.1. In silico mapped contigs were ordered and oriented relative to one another with bridging PCRs and amplicon sequencing by using different primer pairs designed in the present study or previously published (Supplementary Materials Table S3) [62,63]. Insertion sequences (IS) were detected using ISfinder (http://www-is.biotoul.fr, France, accessed on 10 February 2022). *A. baumannii* resistance (*Aba*R) islands were initially identified based on the presence of antimicrobial resistance genes in the genome sequences by using ResFinder. PCR mapping was performed to confirm the genetic arrangements of *Aba*R in case the genome contigs did not comprise the entire island structure. Primers and expected amplicon sequences of mapping PCRs are given in Supplementary Materials Table S5).

### 4.7. Southern Blot Hybridization Analysis

The genomic location of CP genes was verified by S1-Nuclease digestion of whole-cell DNA, pulsed-field gel electrophoresis (PFGE) and Southern hybridisation with DNA probes consisting of a 642-bp internal PCR fragment specific for the *bla*_OXA-23_ gene (primers OXA23-FWD/OXA23-REV), a 456-bp internal PCR fragment specific for the *bla*_OXA-58_ gene (primers OXA58-FWD/OXA58-REV) [13,54] (Supplementary Materials Table S4).

### 4.8. Transformation of Plasmids Carrying Carbapenemase Genes

For transformation of *bla*_OXA-23_ and *bla*_OXA-58_ encoding plasmids of *A. baumannii* isolates electrocompetent cells of *A. baumannii* strain ATCC 17978 were prepared as described previously [62]. Plasmid DNA of donor strains was isolated using the QIAprep Mini-spin kit (Qiagen GmbH, Hilden, Germany). Electroporation was carried out with the Eppendorf Eporator^®^ (Eppendorf, Hamburg, Germany) using the adjustments of 25 µF, 200 Ω and 2.5 kV. The transformants were selected on Mueller–Hinton agar plates containing 4 µg/mL meropenem [62]. The identity of transformants was tested by PCR targeting the CP genes, by PFGE of ApaI-restricted genomes and by phenotypic susceptibility testing using the VITEK 2 system (bioMérieux, Nuertingen, Germany; AST cards GN38 and GN238).

## 5. Conclusions

Here, we describe the occurrence and molecular characteristics of OXA-23- and OXA-58-producing *A. baumannii* isolates obtained from clinical samples of companion animals belonging to the international successful clonal lineages ST1 and ST25. Together with the findings that *bla*_OXA-23_ was integrated into the transposon structures Tn*2006* and Tn*2008* as well as *bla*_OXA-58_ embedded in a previously described genetic context, our data suggest a transfer of acquired carbapenemases and multidrug-resistant *A. baumannii* lineages between humans and companion animals. These findings warrant further investigations on the epidemiology and underlying genetic mechanisms of carbapenem resistance in *Acinetobacter* spp. strains from animals and on the processes that may favour the emergence and spread of such bacteria in veterinary settings.

## Figures and Tables

**Table 1:** Distribution of carbapenemase genes among *A. baumannii* isolates from animal sources.

Sample Collection *	*bla*_OXA-23_	*bla*_OXA-58_	Carbapenemase Positive *Ab* Isolates			
			Host/Origin	Country	IC	ST^Pa^
	*n* (%)	*n* (%)	(*n* Isolates)			
1 (*n* = 473 *Ab* Isolates)	0	15 (3.2)	dog (10), cat (4), air con-ditioner (1)	GER (15)	IC1 (15)	ST1 (15)
2 (*n* = 30 *Ab* Isolates)	13	14	dog/cat (18/10)	GER (16), FRA (9), ITA (3)	IC1 (13), IC7 (10), IC10 (1), n.a. (4)	ST1 (13), ST10 (1), ST25 (10), ST578 (1), ST602 (3)

* Sample collection 1: isolates not pre-selected for susceptibility to carbapenems or any other antibiotic class; sample collection 2: isolates pre-selected for carbapenem non-susceptibility by VITEK 2 (MICs ≥ 2 mg/L for imipenem). Abbreviations: Ab, *A. baumannii* n, number; n.a., not assigned; GER, Germany; FRA, France; ITA, Italy; ST^Pa^, multilocus sequence type according to the Pasteur scheme (https://pubmlst.org/bigsdb?db=pubmlst_abaumannii_pasteur_seqdef; access on 20 May 2022).

**Table 2:** Characteristics of 42 carbapanemase-producing *Acinetobacter baumannii* isolates, sorted by ST^Pa^.

Strain ID	Host	Source	Date of Isolation	Country	IC	ST^Pa^	ST^Ox^	cgMLST		VC ^a^	Acquired OXA ^b^	OXA-Flanking Pattern ^c^	OXA-51 Type
								CT	Cluster				
IHIT28446	Cat	Urine	12/2014	DE	1	1	231	1808	1	1	58^PL^	B	69
IHIT29418	Dog	Wound	06/2015	DE	1	1	231	1808	1	3	58^PL^	C	69
IHIT29480	Cat	CVC	06/2015	DE	1	1	231	1808	1	4	58^PL^	C	69
IHIT29548	Env.	Air cond.	06/2015	DE	1	1	231	1808	1	4	58^PL^	C	69
IHIT29580	Cat	Phlegmon	06/2015	DE	1	1	231	1808	1	4	58^PL^	C	69
IHIT29983	Cat	Nose	06/2015	DE	1	1	231	1808	1	6	58^PL^	D	69
IHIT29985	Dog	Abdomen	03/2015	DE	1	1	231	1808	1	1	58^PL^	D	69
IHIT30000	Dog	Nose	05/2015	DE	1	1	231	1808	1	1	58^PL^	B	69
IHIT33215	Dog	Urine	11/2015	DE	1	1	231	1808	1	6	58^PL^	C	69
IHIT32291	Dog	Wound	12/2015	DE	1	1	231	1808	1	1	58^PL^	B	69
IHIT31605	Dog	Wound	05/2016	DE	1	1	231	1808	1	4	58^PL^	A	69
IHIT31634	Dog	CVC	06/2016	DE	1	1	231	1808	1	4	58^PL^	A	69
IHIT31820	Dog	CVC	06/2016	DE	1	1	231	1808	1	4	58^PL^	A	69
IHIT32293	Dog	Wound	01/2016	DE	1	1	231	1808	1	13	58^PL^	A	69
IHIT32295	Dog	Urine	03/2016	DE	1	1	231	1808	1	14	58^PL^	A	69
IHIT32298	Cat	Urine	05/2016	DE	1	1	231	1808	1	1	58^PL^	A	69
IHIT32299	Cat	Wound	06/2016	DE	1	1	231	1808	1	15	58^PL^	A	69
IHIT33967	Cat	CVC	02/2017	DE	1	1	231	1808	1	4	58^PL^	A	69
IHIT34210	Dog	Throat	04/2017	DE	1	1	231	1808	1	17	58^PL^	B	69
IHIT34211	Dog	Nose	04/2017	DE	1	1	231	2175	S	17	58^PL^	B	69
IHIT34212	Dog	BAL	04/2017	DE	1	1	231	1808	1	17	58^PL^	B	69
IHIT34531	Dog	Wound	05/2017	DE	1	1	231	1808	1	1	58^PL^	A	69
IHIT34607	Dog	Abdomen	05/2017	DE	1	1	231	1808	1	17	58^PL^	B	69
IHIT35448	Cat	Skin	10/2017	DE	1	1	231	1808	1	20	58^PL^	B	69
IHIT36934	Dog	CVC	04/2018	DE	1	1	231	1808	1	4	58^PL^	A	69
IHIT36988	Dog	Wound	03/2018	DE	1	1	231	1808	1	21	58^PL^	B	69
IHIT37071	Dog	BAL	05/2018	DE	1	1	231	1808	1	4	58^PL^	A	69
IHIT38001	Dog	Skin	08/2018	DE	1	1	231	1808	1	22	58^PL^	B	69
IHIT29027	Dog	Urine	07/2015	IT	8	10	447	2190	S	2	23^PL^	b	68
IHIT29982	Dog	Urine	07/2015	FR	7	25	229	2184	3	5	23^CH^	a	64
IHIT29995	Dog	Ear	07/2015	FR	7	25	229	2185	S	7	23^CH^	a	64
IHIT30557	Dog	Urine	09/2015	FR	7	25	229	2184	3	5	23^CH^	a	64
IHIT32250	Cat	Urine	08/2016	FR	7	25	229	2186	3	10	23^CH^	a	64
IHIT32292	Dog	Paw	01/2016	DE	7	25	229	2177	4	12	23^PL^	b	64
IHIT32297	Dog	Trachea	04/2016	DE	7	25	229	2177	4	12	23^PL^	b	64
IHIT32362	Dog	Urine	10/2016	DE	7	25	229	2177	4	16	23^PL^	b	64
IHIT34486	Cat	Wound	05/2017	IT	7	25	229	2182	S	18	23^PL^	c	64
IHIT34502	Cat	Urine	05/2017	FR	7	25	229	2181	S	19	23^PL^	d	64
IHIT29997	Dog	BAL	08/2015	FR	n.t.	578	799	2189	S	9	58^PL^	E	65
IHIT29996	Cat	Tissue	06/2015	FR	n.t.	602	732	1368	2	8	23^PL^	e	378
IHIT30558	Cat	Urine	09/2015	FR	n.t.	602	732	2191	2	8	23^PL^	e	378
IHIT32289	Cat	Urine	01/2016	FR	n.t.	602	732	2191	2	11	23^PL^	e	378

^a^ VC = veterinary clinic; different veterinary clinics are indicated by numbers 1–22. ^b^ Location of gene on plasmid (^PL^) or chromosome (^CH^) is indicated. ^c^: as illustrated in Figure 3 and Figure 4. Abbreviations: n.t., not typeable; Air cond., Air conditioner; BAL, bronchoalveolar lavage; CVC, central venous catheter; Env, environment; DE, Germany; IT, Italy; FR, France; IC, international clone; ST^Pa^, multilocus sequence type according to the Pasteur scheme; ST^Ox^, MLST type according to the Oxford scheme. CT, cluster type; S, singleton.

**Table 3:** Carbapenemase-positive A. *baumannii* from small animals according to the current literature.

Host	Country	Year	Source	CP Type	CR Isolates/Total No. of Isolates	Localization of CP	Carbapenem Resistance	IC	ST^Pa/Ox^	Ref.
Cat	DE	2000	Ur	n.s.	1/52	n.s.	IMP	n.s.	n.s.	[29]
Cat	DE	2000	Ur	OXA-23	1/1	P, Tn*2008*	IMP	1	1/231	[9]
Cat, dog	CH	2005, 2009	Ur, Wo, Bl	IS*Aba1*-OXA-51	2/19	C	IMP, MER	1, 2	12^Ox^ & 15^Ox^	[10]
Cat	PT	2009	Ur	OXA-23	1/1	C, Tn*2006*	IMP, MER	2	2^Past^	[11]
Dog	DE	2011	Ur, Sk	OXA-23	3/223	P, Tn*2008*	IMP, MER	1, 8	10/585	[8]
Cat, dog	FR	2011–2015	Ur	OXA-23	7/41	C	IMP, MER	7	25^Past^	[19]
Cat, dog	IT	2014, 2015	Fe	NDM-1	5/5	C, Tn*125*	IMP, MER	2	2^Past^	[25]
Dog	FR	2015	Or, Re	OXA-23	2/4 CR	n.s.	IMP, MER, DOR	7	25^Past^	[12]
Dog	RS	2016	Ur	OXA-72	1/1	P	IMP, MER	1	1^Past^	[27]
Dog	TH	2017	Ur	OXA-23	1/1	Tn*2006*	IMP, MER	2	2^Past^	[26]
Grey Parrot	LUX	2016	Cho	OXA-72	1/1	P	IMP, MER	n.s.	294^Past^	[30]
Cat	PK	2020	Ur	OXA-23	1/1	n.s.	IMP, MER	2	2^Past^	[28]
Cat	IT	2021	Ur	NDM-1	1/1	C	none	7	25^Past^	[13]

CP: carbapenemase; CR: carbapenem-resistant; IC: international clone, ST: sequence type. CH: Switzerland, DE: Germany, FR: France, IT: Italy, PK: Pakistan, PT: Portugal, RS: Serbia, TH: Thailand. UR: urine, Wo: wound, Bl: blood, Sk: skin, Fe: feces, Or, oral, Re, rectal, Cho, Choane. P: plasmid, C: chromosome, Tn: Transposon. DOR: doripenem, IMP: imipenem, MER: meropenem, n.s.: not specified, Ref.: reference. Multilocus sequence types are provided according to the schemes used in the original publications (^Past^ = Pasteur; ^Ox^ = Oxford).

**Table 4:** Sample collection of *A. baumannii* isolates investigated in this study.

	Collection 1 * (*n* = 473)	Collection 2 * (*n* = 29)
Host/Source		
Dog and cat	351 **	29
Horse	46	0
Rabbit, guinea pig, mouse	15	0
Livestock	50	0
Wild and zoo animals	7	0
Birds	3	0
Environment	1	0
**Sample Source**		
Wound, abscess, fistula	125	7
Respiratory tract, nasopharynx	83	4
Skin, hair	52	3
Urinary tract	42	14
Eye, ear	25	1
Faeces, gastrointestinal tract	18	0
Genital tract	17	0
Other clinical sites	36	0
Organ (after necropsy)	40	0
Catheter, implant, tube	34	0
Air conditioner	1	0
**Country**		
Germany	473	18
France	0	9
Italy	0	2

* Sample collection 1: isolates not pre-selected for susceptibility to carbapenems or any other antibiotic class; sample collection 2: isolates pre-selected for carbapenem non-susceptibility by VITEK 2 (MICs ≥ 2 mg/L for imipenem). ** 34 of 351 isolates were obtained from catheters, implants and tubes.

## Data Availability

All relevant data are provided in the paper and its Supplements. Raw sequence reads of 42 *A. baumannii* genomes are provided under NCBI Bioproject ID PRJNA857701. Further raw data can be made available on reasonable request.

## Supplementary Materials

The following are available online at https://www.mdpi.com/article/10.3390/antibiotics11081045/s1, Figure S1: Neighbor Joining Tree of 28 *Acinetobacter baumannii* isolates based on 2.390 genes included in the cgMLST scheme of Ridom SeqSphere+. Table S1: MICs (mg/L) of *A. baumannii* recipient strain ATCC 17978 after transformation of *bla*_OXA_ plasmids from *A. baumannii*. Table S2: Antimicrobial resistance genes and antimicrobial susceptibility of carbapenemase-producing *A. baumannii* isolates from cats and dogs. Table S3: Primers used for the determination of genetic regions flanking the *bla*_OXA-23_ and *bla*_OXA-58_ genes in *Acinetobacter baumannii*. Table S4: Oligonucleotide primers used for single and multiplex PCRs to detect β-lactamase genes in *Acinetobacter baumannii* isolates. Table S5: Oligonucleotide primers used for PCR mapping of *A. baumannii* resistance island *Aba*R3.
