# Supplementary material for: Genomic Analysis of Acinetobacter baumannii Isolates Carrying OXA-23 and OXA-58 Genes from Animals Reveals ST1 and ST25 as Major Clonal Lineages
Source: Antibiotics (Basel). 2022 Aug 3;11(8):1045. doi: 10.3390/antibiotics11081045 (PMC9404926; doi:10.3390/antibiotics11081045)
Supplement: Supplementary file 1 [file antibiotics-11-01045-s001.zip › Supplemental Figure S1.pdf]

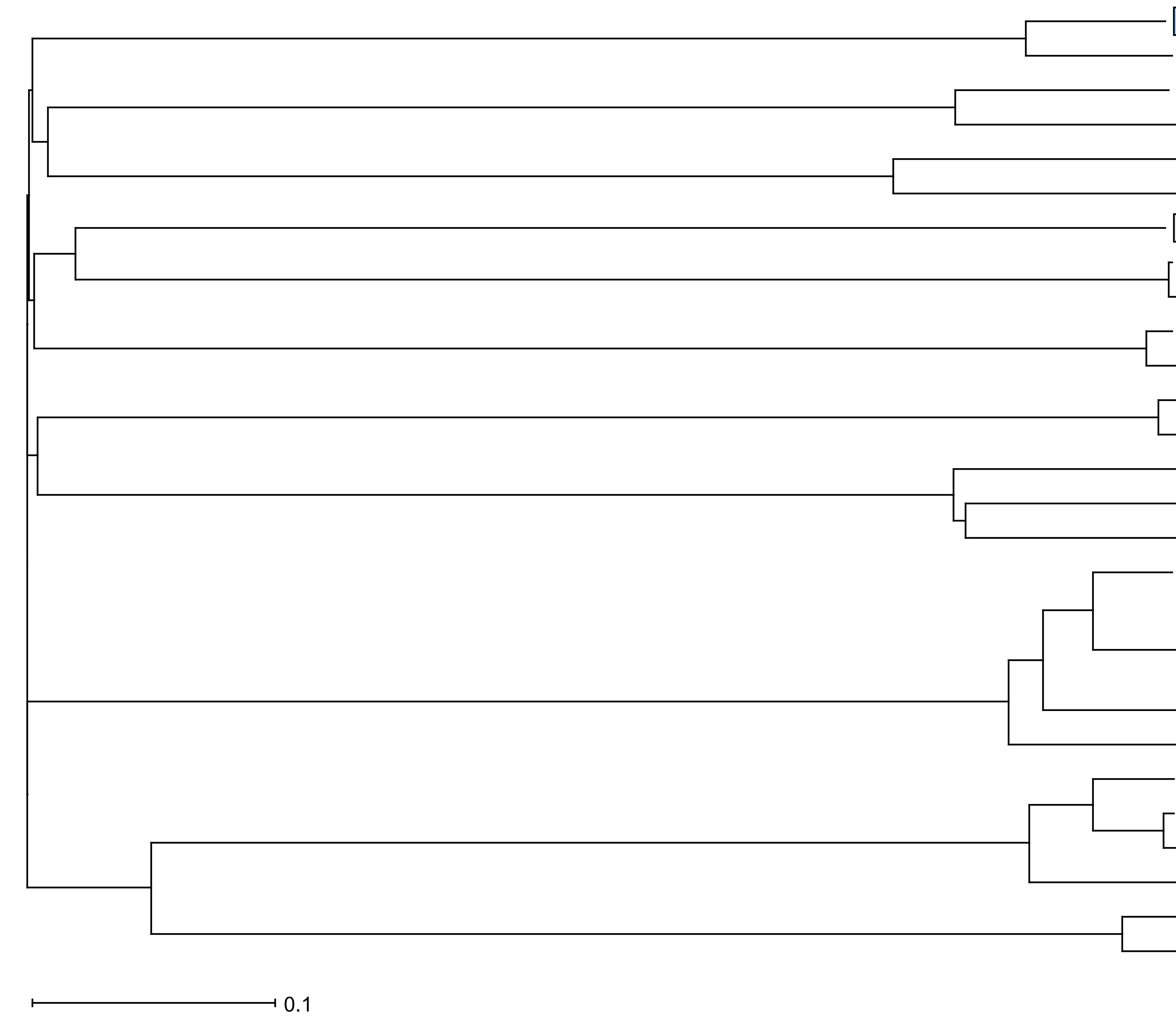

Strain name	International clone	ST^Pasteur	cgMLST complex type	Host	Collection year	Country of origin
AB030	IC5	79	291	Human	2010	Canada
AF-401	IC5	79	1020	Human	2009	Mexico
1656-2	IC2	2	23	Human	2005	South Korea
MDR-TJ	IC2	2	459	Human	2010	China
NIPH 1734	IC4	15	732	Human	2001	Czech Republic
Ab825	IC4	15	3322	Human	1999	Argentinia
IHIT29997	none	578	2189	Dog	2015	France
IHIT32289	none	602	2191	Cat	2016	France
IHIT29996	none	602	1368	Cat	2015	France
Ab-3909	IC6	78	134	Human	2006	n.d.
AB4332	IC6	78	1620	Human	2016	Brazil
MRSN15574	IC9	85	2135	Human	2012	Germany
AC-20	IC9	85	2723	Human	2018	Sudan
XH693	IC8	10	1093	Human	2014	China
IHIT29027	IC8	10	2190	Dog	2015	Italy
LAC-4	IC8	10	9	Human	1997	United States
IHIT32292	IC7	25	2177	Dog	2016	Germany
IHIT32250	IC7	25	2186	Cat	2016	France
IHIT29982	IC7	25	2184	Dog	2015	France
IHIT29995	IC7	25	2185	Dog	2015	France
161/07	IC7	25	22	Human	2007	Germany
LUH6220	IC7	25	452	Human	2000	Netherlands
AYE	IC1	1	7	Human	2001	France
IHIT28446	IC1	1	1808	Cat	2014	Germany
IHIT34211	IC1	1	2175	Dog	2017	Germany
AB0057	IC1	1	290	Human	2004	United States
NIPH 1669	IC3	3	10	Human	1997	Netherlands
Naval-81	IC3	3	475	Human	2006	United States

Supplementary Figure S1: Neighbor Joining Tree of 28 *Acinetobacter baumannii* isolates based on 2.390 genes included in the cgMLST scheme of Ridom SeqSphere+. The tree is based on a comparison of publicly available *A. baumanni* genomes representing international clones IC1 (AYE, GenBank No. NC_010410.1; AB0057, NC011586.2), IC2 (1656-2, NC_017162.1; MDR-TJ, NC_017847.1), IC3 (NIPH 1669, APOQ01.1; Naval-81, AFDB02.1), IC4 (NIPH 1734, APOX01.1, Ab825, NTFR01.1), IC5 (AB030, NZ_CP009257.1; AF-401, NZ_CP018254.1), IC6 (Ab-3909, AEOZ01.1; AB4332, RJLV01.1), IC7 (161/07, JZCA01.1; LUH_6220, JZBW01.1), IC8 (XH693, LYIY01.1; LAC-4, NZ_CP007712) and IC9 (MRSN15574, VHGP01.1; AC-20, JABESZ01.1), and CP positive *A. baumannii* isolates from this study that could not be assigned to a known IC by PCR.
