# Supplementary material for: Genomic Analysis of Acinetobacter baumannii Isolates Carrying OXA-23 and OXA-58 Genes from Animals Reveals ST1 and ST25 as Major Clonal Lineages
Source: Antibiotics (Basel). 2022 Aug 3;11(8):1045. doi: 10.3390/antibiotics11081045 (PMC9404926; doi:10.3390/antibiotics11081045)
Supplement: Supplementary file 1 [file antibiotics-11-01045-s001.zip › Supplemental Table S1.pdf]

Supplemental Table S1: MICs (mg/L) of *A. baumannii* recipient strain ATCC 17978 after transformation of *bla*OXA plasmids from *A. baumannii.*

Strain	Trans-formant	IMP (VITEK)	IMP (E-test)	PIP	CPD	CTF	AMK	GEN	TOB	ENR	MAR	TET	NIT	CHL	PMB	SXT
OXA-23-producing isolates																
IHIT29027	CE-AB12	≥16	≥32	≥128	≥8	≥8	≤2	≤1	≤1	≤0.12	≤0.5	≤1	≥512	32	1	160
IHIT30558	CE-AB18	≥16	≥32	≥128	≥8	≥8	32	≤1	≤1	≤0.12	≤0.5	≤1	≥512	≥64	1	160
IHIT32292	CE-AB14	≥16	≥32	≥128	≥8	≥8	≤2	≤1	≤1	≤0.12	≤0.5	≤1	≥512	≥64	1	160
IHIT32297	CE-AB16	≥16	≥32	≥128	≥8	≥8	≤2	≤1	≤1	≤0.12	≤0.5	≤1	≥512	≥64	1	160
IHIT34486	CE-AB19	≥16	≥32	≥128	≥8	≥8	≤2	≤1	≤1	≤0.12	≤0.5	≤1	≥512	≥64	1	160
OXA-58-producing isolates																
IHIT29480	CE-AB24	≥16	8	≥128	≥8	≥8	≤2	≤1	≤1	≤0.12	≤0.5	≤1	≥512	≥64	1	160
IHIT29985	CE-AB25	≥16	8	≥128	≥8	≥8	≤2	≤1	≤1	≤0.12	≤0.5	≤1	≥512	≥64	1	160
IHIT29997	CE-AB26	8	6	≥128	≥8	≥8	≤2	≤1	≤1	≤0.12	≤0.5	≤1	≥512	≥64	1	160
IHIT31605	CE-AB27	≥16	6	≥128	≥8	≥8	≤2	≤1	≤1	≤0.12	≤0.5	≤1	≥512	≥64	1	160
IHIT31634	CE-AB28	8	3	≥128	4	≥8	≤2	≤1	≤1	≤0.12	≤0.5	≤1	256	≥64	1	160
IHIT31820	CE-AB29	≥16	4	≥128	≥8	≥8	≤2	≤1	≤1	≤0.12	≤0.5	≤1	≥512	≥64	0.5	160
IHIT32291	CE-AB30	≥16	6	≥128	≥8	≥8	≤2	≤1	≤1	≤0.12	≤0.5	≤1	≥512	≥64	1	160
IHIT32293	CE-AB31	8	8	≥128	≥8	≥8	≤2	≤1	≤1	≤0.12	≤0.5	2	256	≥64	1	160
IHIT32295	CE-AB32	8	6	≥128	4	≥8	≤2	≤1	≤1	≤0.12	≤0.5	≤1	256	≥64	0.5	160
IHIT32298	CE-AB33	≥16	8	≥128	≥8	≥8	≤2	≤1	≤1	≤0.12	≤0.5	≤1	≥512	≥64	0.5	160
IHIT32299	CE-AB34	≥16	8	≥128	≥8	≥8	≤2	≤1	≤1	≤0.12	≤0.5	≤1	≥512	≥64	0.5	160
IHIT33967	CE-AB35	≥16	8	≥128	≥8	≥8	≤2	≤1	≤1	≤0.12	≤0.5	≤1	≥512	≥64	0.5	160
IHIT34210	CE-AB36	≥16	8	≥128	≥8	≥8	≤2	≤1	≤1	≤0.12	≤0.5	≤1	≥512	≥64	1	160
IHIT34211	CE-AB37	≥16	6	≥128	≥8	≥8	≤2	≤1	≤1	≤0.12	≤0.5	≤1	≥512	≥64	1	160
IHIT34212	CE-AB38	≥16	6	≥128	≥8	≥8	≤2	≤1	≤1	≤0.12	≤0.5	≤1	≥512	≥64	1	160
IHIT34607	CE-AB39	≥16	6	≥128	≥8	≥8	≤2	≤1	≤1	≤0.12	≤0.5	≤1	≥512	≥64	1	160
**ATCC 17978**	**Recipient**	**≤1**	**≤1**	**16**	**≥8**	**≥8**	**≤2**	**≤1**	**≤1**	**≤0.12**	**≤0.5**	**≤1**	**≥512**	**≥64**	**2**	**160**

MICs were determined with the VITEK 2 system; (MICs for imipenem were additionally determined by antibiotic gradient strips (E-test); all isolates were resistant to ampicillin, amoxicillin/clavulanate, cephalexin, and chloramphenicol in accordance to the definitions of intrinsic resistance for *Acinetobacter* spp. set by EUCAST and CLSI. IMP, imipenem; PIP, piperacillin; CPD, cefpodoxime; CTF, ceftiofur; ENR, enrofloxacin; MAR, marbofloxacin; AMK, amikacin; GEN, gentamicin; TOB, tobramycin; TET, tetracycline; CHL, chloramphenicol; PMB, polymyxin B; SXT, sulfamethoxazole/trimethoprim; NIT, nitrofurantoin.
