# Supplementary material for: Genomic Analysis of Acinetobacter baumannii Isolates Carrying OXA-23 and OXA-58 Genes from Animals Reveals ST1 and ST25 as Major Clonal Lineages
Source: Antibiotics (Basel). 2022 Aug 3;11(8):1045. doi: 10.3390/antibiotics11081045 (PMC9404926; doi:10.3390/antibiotics11081045)
Supplement: Supplementary file 1 [file antibiotics-11-01045-s001.zip › Supplemental Table S3.pdf]

Supplemental Table S3: Primers used for the determination of genetic regions flanking the *bla*$_{OXA-23}$ and *bla*$_{OXA-58}$ genes in *Acinetobacter baumannii*

PCR	Target region	Primer name (Primer ID IHIT)	Primer sequence (5`- 3`)	Amplicon size	Primer reference	OXA region with positive PCR reaction*
			**OXA-23**			
**1**	IS*Aba*1	ISAba1-A_RP	CCACGATAAACGATTGCGAGCA	1.703 bp	PS	a, b, c, d, e
	ATPase	ATPase-univ-R	GCAACCGTCGACATCTGTTA		[1]	
**2**	IS*Aba*1	ISAba1-B_RP	CGGTGAGCAGTGGTTTACAT	1.362 bp	PS	b, c, e
	traL	TraL_RP	CTTCAAGACCGCATCCAACA		PS	
**3**	IS*Aba*1	ISAba1_FWD	TTGGTTGGTCGGGTGAAACT	3.660 bp	PS	e
	korB	KorB_RP	AAGAAGGCGTGGCAGAGAAT		PS	
**4**	*traD*	TraD_Fwd	AGGCCGACTTACCCAATCAC	2.212 bp	PS	e
	Hypothetical protein	HP4_IHIT29996_REV	TTGGCTGCATCATTTTCAGC		PS	
**5**	*Replication protein*	ReplP_FWD	TGGCACTATCATTGCCTGGAC	3.023 bp	PS	b
	strB	StrB_RP	CGATCAAGCAGGTGCGACA		[3]	
**6**	*strA*	StrA-RP	GCAACCAGTCAGAATGCGAT	1.856 bp	[3]	b
	topB	TopB_RP	GGTCATGTTCTGCATATCCTA		PS	
**7**	*ATPase*	ATPase-intern1	TCAGGAAGATCGGACAGATC	1.092 bp (b); 2.504 bp (c); 2.383 bp (d)	[1]	b, c, d
	parB	ParB-RP	CATCTAGGAGAGCTTCGCAA		PS	
**8**	*Hypothetical protein*	HP2_IHIT32289_REV	ATCCGGGTGCATGAGTGAC	2.192 bp	PS	e
	higB2	HigB2_FWD	AACTGGGGCCAAATCTAGGC		PS	
**9**	*dnaJ*	DnaJ_FWD	ATATCTTCAGTCGAGCCGCC	1.604 bp	PS	e
	Hypothetical protein	HP3 IHIT29996_REV	AACGGTGGAGCAGAAGAAAGT		PS	
**10**	IS*Aba*1	ISAba1_FWD	TTGGTTGGTCGGGTGAAACT	3.270 bp	PS	a
	comM	ComM-REV	TTGCCTCTGGTCAACTTCCG		PS	
**11**	*bla*$_{OXA-23}$	OXA-23_FWD	TGTCCTTGAACAATCTGACTC	4.087 bp	[1]	a
	comM	ComM-REV	TTGCCTCTGGTCAACTTCCG		PS	
**12**	*sulP*	SulP-FWD	TCAACGCCTGCAATAATGG	2.258 bp	PS	a
	ATPase	ATPase-intern1	TCAGGAAGATCGGACAGATC		[1]	

PCR	Target region	Primer name (Primer ID IHIT)	Primer sequence (5`- 3`)	Amplicon size	Primer reference	OXA region with positive PCR reaction*
			**OXA-58**			
**13**	*bla*$_{OXA-58}$	OXA-58_FWD	AAGTATTGGGGCTTGTGCTG	2.110 bp (A-D); 3.395 bp (E)	[2]	A, B, C, D
	araC	AraC_REV	CGTAGGGTTGATTCGATAGCA		[3]	
**14**	*adh*	Adh_FWD	ACCAAGATCCGTTCCACAAA	1.412 bp	PS	A, B
	bla$_{OXA-58}$	OXA-58_REV	TCAACTTCCGTGCCTATTTG		[2]	
**15**	*repE*	RepE_FWD	ATGCTACAAGATCCCGCTCA	1.553 bp	PS	A
	adh	Adh_REV	ACGTTGGTGTGCATGGTAAG		PS	
**16**	*adh*	Adh_FWD	ACCAAGATCCGTTCCACAAA	1.536 bp	PS	A
	tonB	TonB_REV	ATTCCGTACCCAAGGCATCC		PS	
**17**	*araC*	AraC_FP	ACAAACACTCTCAACATTGGTG	1.640 bp	[3]	A
	vapC	VapC_REV	CCATAAGCATGGGCACATTGT		PS	
**18**	*tonB*	TonB_FWD	TTACCTCCTGCTCGTTTGGG	958 bp	PS	C
	spl	Spl_REV	ATGCCACTTTATTTCCCGCG		PS	
**19**	*Hypothetical protein*	HP6_IHIT29997_FWD	ACAAGCCTGGAATTAGAGCCA	2.990 bp	PS	D
	bla$_{OXA-58}$	OXA-58_REV	TCAACTTCCGTGCCTATTTG		[2]	
**20**	*repE*	RepE_FWD	ATGCTACAAGATCCCGCTCA	2.730 bp	PS	C
	bla$_{OXA-58}$	OXA-58_REV	TCAACTTCCGTGCCTATTTG		[2]	
**21**	*Hypothetical protein*	HP3_ IHIT29985_FWD	ACCTCATCTTTAGAAGCGCCA	699 bp	PS	C
	open reading frame	ORF_IHIT29985_REV	TTGTCACACACCACGCATCT		PS	

* see Figures 2 and 3 of the main text for genetic structures of OXA regions; PS = present study

Primer references:

1. Klotz P, Goettig S, Leidner U, Semmler T, Scheufen S, Ewers C. 2017. Carbapenem-resistance and pathogenicity of bovine *Acinetobacter indicus*-like isolates. PLOS ONE 12(2):e0171986. doi: 10.1371/journal.pone.0171986
2. Groebner S, Linke D, Schutz W, Fladerer C, Madlung J, Autenrieth IB, Witte W, Pfeifer Y. 2009. Emergence of carbapenem-non-susceptible extended-spectrum β-lactamase-producing *Klebsiella pneumoniae* isolates at the university hospital of Tubingen, Germany. Journal of Medical Microbiology **58**:912–922. doi: 10.1099/jmm.0.005850-0.
3. Klotz P, Jacobmeyer L, Leidner U, Stamm I, Semmler T, Ewers C. 2018. *Acinetobacter pittii* from Companion Animals Coharboring *bla*$_{OXA-58}$, the *tet*(39) Region, and Other Resistance Genes on a Single Plasmid. Antimicrobial Agents and Chemotherapy 62(1):e01993-17. doi: 10.1128/AAC.01993-17.

Supplemental Table S3 continued:

According to the different genetic environments of *bla*$_{OXA-23}$ and *bla*$_{OXA-58}$ genes (see Figure 2 and Figure 3 of the main text), *bla*$_{OXA-23}$ and *bla*$_{OXA-58}$-positive *A. baumannii* strains revealed different PCR results:

		**PCR numbers (positive PCR results are indicated by blue-filled boxes)**																				
**OXA gene flanking pattern**	**Location of *bla*$_{OXA}$ gene and/or plasmid size**	1	2	3	4	5	6	7	8	9	10	11	12	13	14	15	16	17	18	19	20	21
		**OXA-23**																				
a	chromosome	■									■	■	■									
b	48.4 kb	■	■			■	■	■														
c	55.8 kb	■	■					■														
d	unknown	■	■					■														
e	71.3 kb	■		■	■				■	■												
		**OXA-58**																				
A	21.1 kb													■	■	■	■	■				
B	11.3 kb													■	■							
C	12.6 kb													■							■	
D	12.3 kb													■					■			■
