# Supplementary material for: Genomic Analysis of Acinetobacter baumannii Isolates Carrying OXA-23 and OXA-58 Genes from Animals Reveals ST1 and ST25 as Major Clonal Lineages
Source: Antibiotics (Basel). 2022 Aug 3;11(8):1045. doi: 10.3390/antibiotics11081045 (PMC9404926; doi:10.3390/antibiotics11081045)
Supplement: Supplementary file 1 [file antibiotics-11-01045-s001.zip › Supplemental Table S4.pdf]

Supplemental Table S4: Oligonucleotide primers used for single and multiplex PCRs to detect β-lactamase genes in *Acinetobacter baumannii* isolates

Target gene	Primer name	Primer sequence (5`- 3`)	Amplicon size	Primer reference
bla_{OXA-23}	OXA-23 fwd	AACCCCGAGTCAGATTGTTC	642 bp	[1]
	OXA-23 rev	ATTTTTCCATCTGGCTGCTC		
bla_{OXA-40}	OXA-40 fwd	GTTTCTCTCAGTGCATGTTCATC	246 bp	[1]
	OXA-40 rev	CGAATAGAACCAGACATTCCTTC		
bla_{OXA-58}	OXA-58 fwd	AAGTATTGGGGCTTGTGCTG	599 bp	[1]
	OXA-58 rev	TCAACTTCCGTGCCTATTTG		
$bla_{OXA-143}$	OXA-143 fwd	TGGCACTTTCAGCAGTTCCT	149 bp	[2]
	OXA-143 rev	TAATCTTGAGGGGGCCAACC		
$bla_{OXA-235}$	OXA-235 fwd	TTGTTGCCTTTACTTAGTTGC	768 bp	[3]
	OXA-235 rev	CAAAATTTTAAGACGGATCG		
bla_{NDM}	NDM-1_F	CTGAGCACCGCATTAGCC	754 bp	[4]
	NDM-1_R	GGGCCGTATGAGTGATTGC		
bla_{KPC}	KPC_F	CAGCTCATTCAAGGGCTTTC	533 bp	[1]
	KPC_R	AGTCATTTGCCGTGCCATAC		
bla_{VIM}	VIM_F	AGTGGTGAGTATCCGACAG	261 bp	[1]
	VIM_R	ATGAAAGTGCGTGGAGAC		
bla_{OXA-48}	OXA-48A	TTGGTGGCATCGATTATCGG	743 bp	[5]
	OXA-48B	GAGCACTTCTTTTGTGATGGC		
bla_{GES}	GES_Fwd	GAAGCTAGAGCGCGAAAAAG	744 bp	[6]
	GES_Rev	ACTTGACCGACAGAGGCAAC		
bla_{GIM}	GIM-ges F	TGTAGCGTTGCCAGCTTTAG	700 bp	[7]
	GIM-ges R	GGTCGGTTGCATTAATTTGTTAG		
bla_{IMP}	IMP_F	GGAATAGAGTGGCTTAAYTCTC	233 bp	[8]
	IMP_R	GGTTTAAYAAAACAACCACC		

Primer references

1. Groebner, S.; Linke, D.; Schutz, W.; Fladerer, C.; Madlung, J.; Autenrieth, I.B.; Witte, W.; Pfeifer, Y. Emergence of carbapenem-non-susceptible extended-spectrum β-lactamase-producing *Klebsiella pneumoniae* isolates at the university hospital of Tübingen, Germany. Journal of medical microbiology 2009, 58, 912-922, doi:10.1099/jmm.0.005850-0.
2. Higgins, P.G.; Lehmann, M.; Seifert, H. Inclusion of OXA-143 primers in a multiplex polymerase chain reaction (PCR) for genes encoding prevalent OXA carbapenemases in *Acinetobacter* spp. International journal of antimicrobial agents 2010, 35, 305, doi:10.1016/j.ijantimicag.2009.10.014.
3. Higgins, P.G.; Perez-Llarena, F.J.; Zander, E.; Fernandez, A.; Bou, G.; Seifert, H. OXA-235, a novel class D β-lactamase involved in resistance to carbapenems in *Acinetobacter baumannii*. Antimicrobial agents and chemotherapy 2013, 57, 2121-2126, doi:10.1128/AAC.02413-12.
4. Pfeifer, Y.; Witte, W.; Holfelder, M.; Busch, J.; Nordmann, P.; Poirel, L. NDM-1-producing *Escherichia coli* in Germany. Antimicrobial agents and chemotherapy 2011, 55, 1318-1319, doi:10.1128/AAC.01585-10.
5. Poirel, L.; Dortet, L.; Bernabeu, S.; Nordmann, P. Genetic features of bla_{NDM-1}-positive Enterobacteriaceae. Antimicrobial agents and chemotherapy 2011, 55, 5403-5407, doi:10.1128/AAC.00585-11.

6. Eller, C. Molecular analysis of extended-spectrum β-lactamase (ESBL)-producing Enterobacteriaceae as colonisers and pathogens in humans. 2014. Dissertation; Faculty of Life Sciences, Technical University Braunschweig, Germany. doi: 10.24355/dbbs.084-201411260838-0.
7. Pfeifer, Y., Robert-Koch Institute Wernigerode, Germany; personal communication
8. Poirel, L.; Walsh, T.R.; Cuvillier, V.; Nordmann, P. Multiplex PCR for detection of acquired carbapenemase genes. Diagnostic microbiology and infectious disease 2011, 70, 119-123, doi:10.1016/j.diagmicrobio.2010.12.002.
