# Supplementary material for: Genomic Analysis of Acinetobacter baumannii Isolates Carrying OXA-23 and OXA-58 Genes from Animals Reveals ST1 and ST25 as Major Clonal Lineages
Source: Antibiotics (Basel). 2022 Aug 3;11(8):1045. doi: 10.3390/antibiotics11081045 (PMC9404926; doi:10.3390/antibiotics11081045)
Supplement: Supplementary file 1 [file antibiotics-11-01045-s001.zip › Supplemental Table S5.pdf]

Supplemental Table S5: Oligonucleotide primers used for PCR mapping of *A. baumannii* resistance island *Aba*R3

PCR No.	Primer-Nr.	Target gene	Target region	Primer name	5´-3´sequence	Position in *Aba*R3 of *A. baumannii* A85 (GenBank Acc-No. KC118540)	Amplicon size in strain A85*	Primer reference
1	1898	cadR	*Aba*R3	AbaR3-FP1	ATCTGCGAGCGTACATGAG	59603 - 59621	3.959 bp	This study
	1900	topA	*Aba*R3	AbaR3-RP1	TGATCTTCTTCACCTTGCC	63543 - 63561		
2	326	$bla_{TEM\text{-}like}$	*Aba*R3	TEM-FW	ATGAGTATTCAACATTTCCG	86241 - 86260	1.778 bp	[1]
	1901	tnpA	*Aba*R3	AbaR3-RP2	TGTAAAAACGGATGAATGAGG	87998 - 88018		
3	1899	tnpR	*Aba*R3	AbaR3-FP2	TCAGAGACCATGGAAAGCATG	91477 - 91497	3.861 bp	This study
	1902	xerD, intI1	*Aba*R3	AbaR3-RP3	TAGAACAAGCAGGCATCACG	95318 - 95337		
4	1898	cadR	*Aba*R3	AbaR3-FP1*	ATCTGCGAGCGTACATGAG	102879 - 102897	3.207 bp	This study
	1903	uspA	*Aba*R3	AbaR3-RP4	TTCAGTCTGGATGCTCATAG	106066 - 106085		
5	22	aadA1	*Aba*R3	aadA1-FOR	ATGAGGGAAGCGGTGATCG	97809 - 97827	3.484 bp	[2]
	1904	resX	*Aba*R3	AbaR3-RP5	TCCGCCGGGCAAGGTCATTTC	101272 - 101292		This study
6	1905	aacC1	*Aba*R3	AbaR3-FP3	TGACGATCCCGCAGTGGCTC	96678 - 96697	3.456 bp	This study
	1906	orf5	*Aba*R3	AbaR3-RP6	ACAACCTCGTCGATATCACC	100114 - 100133		
7	1905	aacC1	*Aba*R3	AbaR3-FP3	TGACGATCCCGCAGTGGCTC	96678 - 96697	4.615 bp	This study
	1904	resX	*Aba*R3	AbaR3-RP5	TCCGCCGGGCAAGGTCATTTC	101272 - 101292		

* The ST1-OXA-58 producing *A. baumannii* isolates from our study that were positive for *Aba*R3-island genes revealed the expected amplicon sizes in all PCR except for PCR no. 3. Our isolates lacked the kanamycin gene *aphA1* and part of its flanking IS*Aba1* regions and were thus about 1.416 bp smaller in size (= 2.445 bp) compared to reference strain A85 (=3.861 bp).

Primer references

1. Grimm, V.; Ezaki, S.; Susa, M.; Knabbe, C.; Schmid, R.D.; Bachmann, T.T. Use of DNA microarrays for rapid genotyping of TEM beta-lactamases that confer resistance. *Journal of clinical microbiology* **2004**, *42*, 3766-3774, doi:10.1128/JCM.42.8.3766-3774.2004.
2. Hujer, K.M.; Hujer, A.M.; Hulten, E.A.; Bajaksouzian, S.; Adams, J.M.; Donskey, C.J.; Ecker, D.J.; Massire, C.; Eshoo, M.W.; Sampath, R.; et al. Analysis of antibiotic resistance genes in multidrug-resistant *Acinetobacter* sp. isolates from military and civilian patients treated at the Walter Reed Army Medical Center. *Antimicrobial agents and chemotherapy* **2006**, *50*, 4114-4123, doi:10.1128/AAC.00778-06.
